# Vascular endothelial growth factor receptor-1 mRNA overexpression in peripheral blood as a useful prognostic marker in breast cancer

**DOI:** 10.1186/bcr3345

**Published:** 2012-10-31

**Authors:** Yoshimasa Kosaka, Akemi Kataoka, Hiroshi Yamaguchi, Hiroaki Ueo, Sayuri Akiyoshi, Norihiko Sengoku, Masaru Kuranami, Shinji Ohno, Masahiko Watanabe, Koshi Mimori, Masaki Mori

**Affiliations:** 1 Department of Surgery, Medical Institute of Bioregulation, Kyushu University, 4546, Tsurumihara, Beppu 874-0838, Japan; 2 Department of Breast Oncology, Kyushu Cancer Center, 3-1-1 Notame, Minami-ku 811-1395, Japan; 3 Ueo Breast Surgical Hospital, 188-2 Haya, Oita 870-0854, Japan; 4 Department of Surgery, Kitasato University School of Medicine, 1-15-1 Kitasato, Minami-ku, Sagamihara 252-0374, Japan

## Abstract

**Introduction:**

Identification of useful markers associated with poor prognosis in breast cancer patients is critically needed. We previously showed that expression of vascular endothelial growth factor receptor-1 mRNA in peripheral blood may be useful to predict distant metastasis in gastric cancer patients. However, expression of vascular endothelial growth factor receptor-1 mRNA in peripheral blood of breast cancer patients has not yet been studied.

**Methods:**

Real-time reverse transcriptase-PCR was used to analyze vascular endothelial growth factor receptor-1 mRNA expression status with respect to various clinical parameters in 515 patients with breast cancer and 25 controls.

**Results:**

Expression of vascular endothelial growth factor receptor-1 mRNA in peripheral blood was higher in breast cancer patients than in controls. Increased vascular endothelial growth factor receptor-1 mRNA expression was associated with large tumor size, lymph node metastasis and clinical stage. Patients with high vascular endothelial growth factor receptor-1 mRNA expression also experienced a poorer survival rate than those with low expression levels, including those patients with triple-negative type and luminal-HER2(-) type disease.

**Conclusions:**

Expression of v*ascular endothelial growth factor receptor-1 *mRNA in peripheral blood may be useful for prediction of poor prognosis in breast cancer, especially in patients with triple-negative type and luminal-HER2(-) type disease.

## Introduction

Breast cancer is the most common malignancy among woman and is one of the major causes of death. During the last several decades, many studies have been conducted to identify markers that indicate breast cancer recurrence and/or prognosis, including disseminated tumor cells (DTCs) and circulating tumor cells (CTCs) [1,2]. However, no precise markers for prediction of relapse-free survival (RFS) and/or overall survival (OS) in patients with breast cancer have yet been identified. Quantitative techniques, such as reverse transcriptase (RT)-PCR, are not currently performed to identify recurrence and survival factors for breast cancer in a daily procedure. Although the high sensitivity of RT-PCR can result in false positives, its power of detection is more sensitive than that of immunohistochemistry.

Recently, Kaplan *et al*. reported that bone marrow derived cells (BMDCs) expressing vascular endothelial growth factor receptor-1 (VEGFR-1) play an important role in the development of malignant metastasis [3]. These investigators showed that BMDCs form a "metastatic niche" in the lungs prior to the arrival of cancer cells, and that blockade of VEGFR-1 prevented this BMDC infiltration and metastatic niche formation. Their findings suggest that VEGFR-1-expressing cells in the bone marrow or peripheral blood may contribute to cancer metastasis and recurrence.

Our previous study demonstrated that *VEGFR-1 *mRNA expression in the peripheral blood of gastric cancer patients is associated with pathological stage and recurrence. However, no reports have yet evaluated the clinicopathologic significance or prognostic value of peripheral blood *VEGFR-1 *mRNA expression in breast cancer patients. Therefore, the aim of the current study was to evaluate the expression of *VEGFR-1 *mRNA in the peripheral blood of >500 breast cancer patients and to define its clinicopathologic and prognostic significance with respect to recurrence and survival.

## Materials and methods

### Patients

This study enrolled 515 breast cancer patients with stage 0 to III disease who underwent surgery in the National Kyushu Cancer Center Hospital, Japan between 2000 and 2004. In addition, 25 patients with no history of cancer who underwent abdominal surgery between 2001 and 2004 were recruited as negative controls; this group included 16 cases of gallstones, 3 cases of common bile duct stones and 6 cases of incisional hernia that occurred following curative surgery more than five years previously. The mean postoperative period was 44.4 months (range, 4 to 86 months). Clinical stages and pathological features of primary tumors were defined according to the classification of the International Union Against Cancer [4]. Patient ages ranged from 28 to 85 years. All of the women included provided written informed consent for participation in the study, and the ethics committees of the Kyushu University and National Kyushu Cancer Center Hospital, Japan, approved the research project.

### Blood sampling

Aspiration of peripheral blood was conducted under general anesthesia immediately prior to surgery. Peripheral blood was obtained through a venous catheter. The first 1 mL of peripheral blood was discarded to avoid contamination by epidermal cells. A 1 mL sample of peripheral whole blood from each patient was immediately mixed vigorously with 4 mL ISOGEN-LS (NIPPON GENE, Toyama, Japan) and stored at -80°C until RNA extraction.

### Total RNA extraction and first-strand cDNA synthesis

Total RNA was extracted according to the ISOGEN-LS manufacturer's protocols. All of the clinical samples obtained from the National Kyushu Cancer Center Hospital were sent to our institute. RT reactions were performed as described previously [5,6]. First-strand cDNA was synthesized from 2.7 µg total RNA in a 30 µL reaction mixture containing 5 µL 5X RT reaction buffer (Gibco BRL, Gaithersburg, MD, USA), 200 µM dNTPs, 100 µM solution random hexadeoxynucleotide primer mixture, 50 units RNasin (Promega, Madison, WI, USA), 2 µL 0.1 M dithiothreitol, and 100 units Moloney murine leukemia virus RT (BRL). The mixture was incubated at 37°C for 60 minutes, heated to 95°C for 10 minutes, and subsequently chilled on ice.

### Quantitative RT-PCR

The following *VEGFR-1 *mRNA primers were used: sense primer, 5'-TCATGAATGTTTCCCTGCAA-3'; antisense primer, 5'-GGAGGTATGGTGCTTCCTGA-3'. Ribosomal protein S27a (RPS27A) was used as an internal control. The following RPS27A primers were used: sense primer, 5'-TCGTGGTGGTGCTAAGAAAA-3'; antisense primer, 5'-TCTCGACGAAGGCGACTAAT-3'. Real-time monitoring of PCR reactions was performed using the LightCycler™ system (Roche Applied Science, Indianapolis, IN, USA) and SYBR green I dye (Roche Diagnostics). Monitoring was performed according to the manufacturer's instructions, as described previously [7,8]. In brief, a master mixture was prepared on ice, containing 500 ng cDNA of each gene, 2 µL LC DNA Master SYBR green I mix, 50 ng primers and 2.4 µL 25 mM MgCl_2_. The final volume was then adjusted to 20 µL with water. After the reaction mixture was loaded into the glass capillary tube, PCR was conducted under the following cycling conditions: initial denaturation at 95°C for 10 minutes, followed by 40 cycles of denaturation at 95°C for 8 sec, annealing at 68°C for 8 sec and extension at 72°C for 2 sec. After amplification, the products were subjected to a temperature gradient from 72°C to 95°C at 0.1°C/sec, under continuous fluorescence monitoring to produce a melting curve of the products.

### Data analysis

Expression levels of *VEGFR-1 *and *RPS27A *mRNAs were determined by comparison with the cDNA from Human Universal Reference Total RNA (Clontech, Palo Alto, CA, USA). After proportional baseline adjustment, the fit point method was employed to determine the cycle in which the log-linear signal was first distinguishable from baseline, and then that cycle number was used as a crossing-point value. The standard curve was produced by measuring the crossing point of each standard value and plotting it against the logarithmic value of the concentrations. Concentrations were calculated by plotting their crossing points against the standard curve, and were adjusted by *RPS27A *content. Taking into consideration the clinical application of the current study, the 95% confidence interval (CI) was used as the upper limit of a normal case cutoff value (bone marrow, 0.12; peripheral blood, 0.059). The 95% value of a normal case according to the reference intervals of the Clinical and Laboratory Standards Institute [9] was established, and the reference limit was regarded as the cutoff value. Levels higher or lower than the cutoff value were considered "positive" and "negative," respectively. The sensitivity and specificity of the data were determined to evaluate the legitimacy of the cutoff value.

### Statistics

For continuous variables, the data were expressed as mean ± standard deviation. The relationships between *VEGFR-1 *mRNA expression and clinicopathological factors were analyzed using the chi-square test and Kruskal-Wallis test. The Kaplan-Meier method was used to estimate the occurrence probability of an event, where the events included death, relapse, local recurrence and observation of distant metastasis. The generalized log-rank test was applied to compare the occurrence probability. Cox proportional-hazards models were fitted for multivariate analysis. All tests were analyzed using JMP software (SAS Institute, Inc., Cary, NC, USA). Statistical significance was determined as a *P*-value from two-sided tests of <0.05.

## Results

### Patient characteristics

Blood samples from 25 normal control cases and 515 breast cancer patients were analyzed for *VEGFR-1 *and *RPS27A *gene expression levels by quantitative real-time RT-PCR; expression levels of *VEGFR-1 *mRNA were corrected for those of *RPS27A *mRNA. An overview of patient characteristics is provided in Table 1.

**Table 1:** Clinicopathologic characteristics of the patients

Factors	**No**.	%
Patients enrolled	515	100.0
Age (mean ± SD)	55.5 ± 11.5	
Tumor size		
Tis	6	1.2
T0	3	0.6
T1	193	37.5
T2	272	52.8
T3	34	6.6
T4	7	1.3
Lymphnode metastasis		
negative	342	66.4
positive	173	33.6
Lymphatic involvement		
negative	316	61.4
positive	199	38.6
Vascular involvement		
negative	481	93.4
positive	34	6.6
Nuclear grade		
I/II	346	67.2
III	169	32.8
Hormone receptor		
negative	123	23.9
positive	392	76.1
HER-2/NEU		
negative	319	61.9
positive	50	9.7
unknown	146	28.4
CEA		
<5.0	452	87.8
>5.1	24	4.7
unknown	39	7.5
Stage		
0	6	1.2
I	155	30.1
II	316	61.3
III	38	7.4
Recurrence		
negative	438	85.0
positive	77	15.0
Prognosis		
alive	488	94.8
dead	27	5.2

SD, standard deviation

### Expression of *VEGFR-1 *mRNA in peripheral blood of surgical breast cancer patients

**Figure 1:**
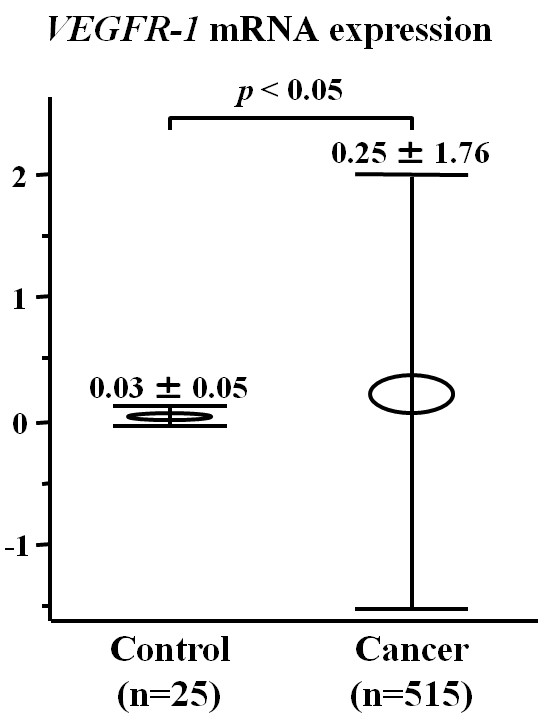
***VEGFR-1 *mRNA expression in peripheral blood from breast cancer patients**. Expression of *VEGFR-1 *mRNA is significantly elevated in peripheral blood in 515 patients of breast cancer in comparison to that in 25 normal control patients.

**Figure 2:**
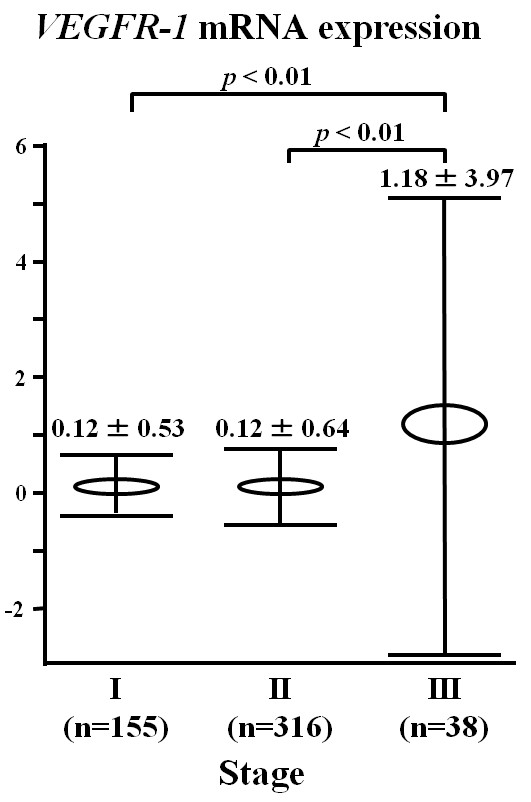
***VEGFR-1 *mRNA expression in clinical stage of breast cancer patients**. The higher *VEGFR-1 *mRNA expression was observed in patients with stage III in comparison with stage I and II patients (*P *<0.01, respectively).

Figure 1 shows peripheral blood *VEGFR-1 *mRNA levels of breast cancer patients and controls. The mean expression level of *VEGFR-1 *mRNA in cancer patients (0.25 ± 1.76) was significantly higher than that of the control group (0.03 ± 0.05; *P *<0.05). Expression levels of *VEGFR-1 *mRNA in stage III breast cancer patients (1.18 ± 3.97) were significantly higher than those of either stage I (0.12 ± 0.53; *P *<0.01) or stage II (0.12 ± 0.64; *P *<0.01) patients (Figure 2).

### Association between *VEGFR-1 *mRNA expression level and clinicopathological features

Correlations between *VEGFR-1 *mRNA levels and clinicopathological variables are summarized in Table 2. By the predetermined cutoff value (0.059), 49 patients (9.52%) were positive for *VEGFR-1 *mRNA expression. Significantly higher expression was observed in the following patient subgroups: large tumor size (*P *<0.05), lymph node metastasis (*P *<0.05), clinical stage (*P *<0.01), postoperative recurrence (*P *<0.01) and poor prognosis (*P *<0.0001).

**Table 2:** Correlation of clinicopathologic characterestics and *VEGFR1 *mRNA expression

	*VEGFR1 *mRNA in peripheral blood				
	
	negative		Positive		
	(n = 466)		(n = 49)		
			
Factors	**No**.	%	**No**.	%	*P*
Age (mean ± SD)	55.5 ± 11.5		54.5 ± 11.5		0.66
Tumor size					
small (Tis to T1)	189	40.6	13	26.5	<0.05
large (T2 to T4)	277	59.4	36	73.5	
Lymphnode metastasis					
negative	316	67.8	26	53.1	<0.05
positive	150	32.2	23	46.9	
Lymphatic involvement					
negative	289	62.0	27	55.1	0.34
positive	177	38.0	22	44.9	
Vascular involvement					
negative	438	94.0	43	87.8	0.09
positive	28	6.0	6	12.2	
Nuclear grade					
I/II	311	66.7	35	71.4	0.50
III	155	33.3	14	28.6	
Hormone receptor					
negative	112	24.0	11	22.4	0.80
positive	354	76.0	38	77.6	
HER-2/NEU					
negative	301	86.0	18	94.7	0.23
positive	49	14.0	1	5.3	
CEA					
<5.0	408	94.9	44	95.7	0.82
>5.1	22	5.1	2	4.3	
Stage					
0	6	1.3	0	0.0	<0.01
I	143	30.7	12	24.5	
II	290	62.2	26	53.1	
III	27	5.8	11	22.4	
Recurrence					
negative	404	86.7	34	69.4	<0.01
positive	62	13.3	15	30.6	
Prognosis					
alive	449	96.4	39	79.6	<0.0001
dead	17	3.6	10	20.4	

### Association between *VEGFR-1 *mRNA expression level and survival

**Figure 3:**
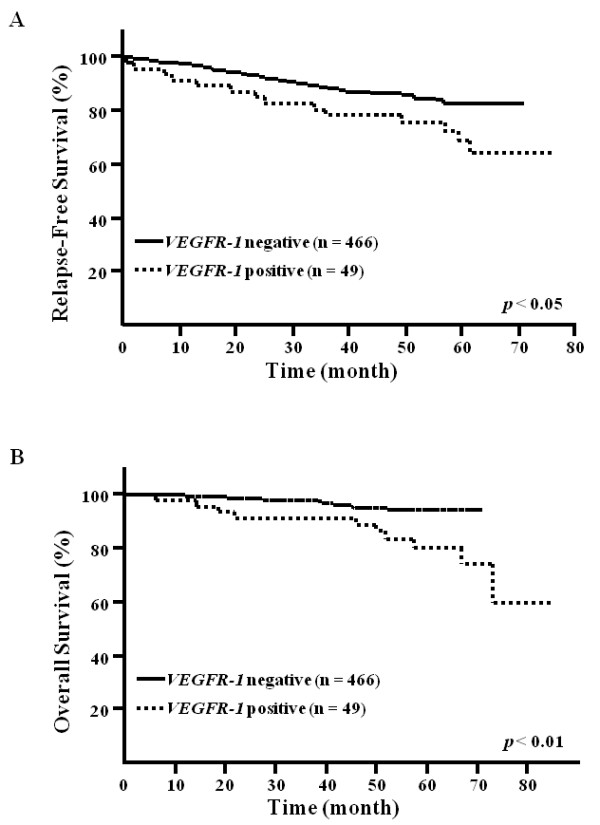
**Kaplan-Meier relapse free survival (A) and overall survival (B) curves**. Patients in the high expression group of *VEGFR-1 *mRNA showed significantly high recurrence (*P *<0.05) and poorer prognosis (*P *<0.01) than those in the low-expression group (log rank test).

At a median follow-up of 44.4 months, 76 RFS events and 27 OS events had been registered. The five-year RFS rate (69.0%) was significantly lower in *VEGFR-1*-positive patients than in *VEGFR-1*-negative patients (82.5%; *P *<0.05; Figure 3A). The five-year OS rate (80.5%) was also significantly lower in *VEGFR-1*-positive patients than in *VEGFR-1*-negative patients (94.4%; *P *<0.01; Figure 3B).

Univariate and multivariate analyses were performed to identify factors associated with survival (Table 3). Univariate analysis identified lymph node metastasis, hormone receptor status, elevated preoperative CEA level (>5.1 ng/mL), tumor size, nuclear grade, lymphatic involvement and *VEGFR-1 *mRNA expression levels as adverse prognostic factors for OS (*P *<0.05 for each factor). Multivariate analysis also indicated that high *VEGFR-1 *mRNA expression was an independent factor for OS (relative risk (RR), 2.00; 95% CI, 1.19 to 3.25, *P *<0.01), as well as lymph node metastasis (RR, 2.67; 95% CI, 1.53 to 4.96, *P *<0.001) and hormone receptor status (RR, 2.65; 95% CI, 1.62 to 4.43, *P *<0.001).

**Table 3:** Univariate and multivariate analysis for overall survival

	Univariate analysis			Multivariate analysis		
		
Factors	RR	95% CI	*P*	RR	95% CI	*P*
Lymphnode metastasis (negative/positive)	2.91	1.86 to 5.03	<0.0001	2.67	1.53 to 4.96	<0.001
Hormone receptor (positive/negative)	2.37	1.61 to 3.59	<0.0001	2.65	1.62 to 4.43	0.0001
*VEGFR-1 *mRNA expression (negative/positive)	1.86	1.19 to 2.79	<0.01	2.00	1.19 to 3.25	<0.01
CEA (<5.0 ng/ml/ >5.1 ng/ml)	2.23	1.20 to 3.65	<0.05	1.69	0.87 to 2.95	0.11
Tumor size (small/large)	2.14	1.26 to 4.39	<0.01	1.63	0.85 to 4.13	0.16
Nuclear grade (I and II/III)	1.62	1.11 to 2.39	<0.05	1.25	0.82 to 1.89	0.29
Lymphatic involvement (negative/positive)	1.51	1.03 to 2.26	<0.05	0.99	0.62 to 1.61	0.96
Vascular involvement (negative/positive)	1.85	1.00 to 3.01	0.05	-	-	-
HER-2/NEU (negative/positive)	1.48	0.96 to 2.26	0.08	-	-	-

CI, Confidence interval; RR, Relative risk

### Association between *VEGFR-1 *mRNA expression level and survival by subtype of breast cancer

**Figure 4:**
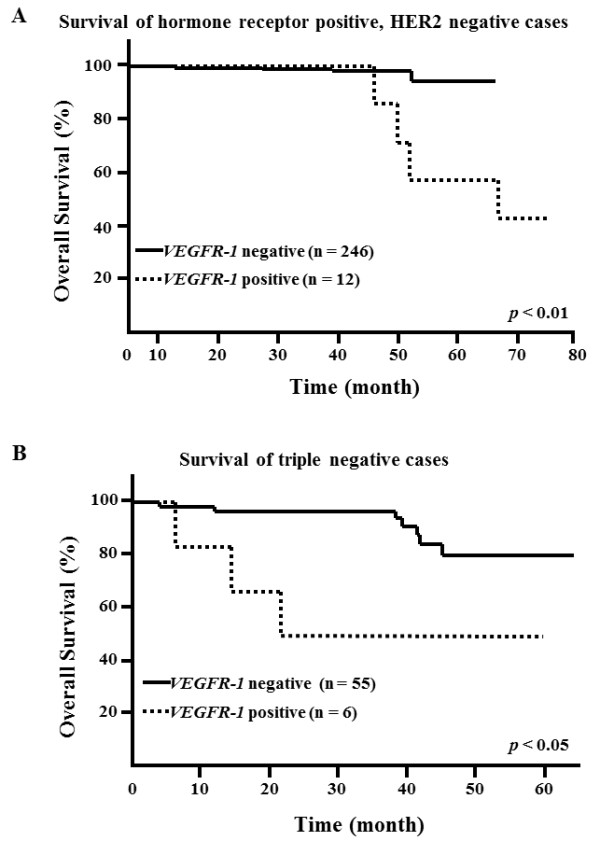
**Kaplan-Meier overall survival curves by subtype of breast cancer**. **(A) **Hormone receptor-positive, HER2-negative breast cancer patients with high expression group of *VEGFR-1 *mRNA showed significantly poorer prognosis (*P *<0.01, log rank test). **(B) **Triple negative breast cancer patients with high expression group of *VEGFR-1 *mRNA showed significantly poorer prognosis (*P *<0.05, log rank test).

In patients with hormone receptor-positive, HER2-negative, the five-year OS rate (57.1%) was significantly lower in *VEGFR-1*-positive patients (n = 12) compared to that in *VEGFR-1*-negative patients (n = 246; 94.2%; *P *<0.01; Figure 4a).

In patients with triple-negative breast cancer (that is, cancers that were estrogen receptor-negative, progesterone receptor-negative and HER2-negative), the five-year OS rate (50%) was significantly lower in *VEGFR-1*-positive patients (n = 6) compared to that in *VEGFR-1*-negative patients (n = 55; 79.9%; *P *<0.05; Figure 4b).

However, there was no significant difference between *VEGFR-1*-positive and *VEGFR-1*-negative in patients with HER2-positive regardless of the presence of the hormone receptor (data not shown).

## Discussion

We have investigated the correlation between *VEGFR-1 *mRNA expression in peripheral blood of a large number of breast cancer patients and clinicopathologic factors, RFS and OS. The present results reveal that *VEGFR-1 *mRNA expression levels are very high in breast cancer patients and are associated with clinical stage, recurrence and prognosis. In addition, *VEGFR-1 *mRNA expression levels are associated with tumor size and lymph node metastasis (Table 2). These results are consistent with our previous reports, in which peripheral blood *VEGFR-1 *mRNA expression levels were significantly higher in gastric cancer patients compared to non-cancer patients, and *VEGFR-1 *mRNA expression levels were associated with clinical stage [10,11].

In the present study, patients with *VEGFR-1 *mRNA overexpression experienced a higher rate of recurrence and a poorer prognosis compared to *VEGFR-1-*negative patients. Moreover, Kaplan-Meier analysis indicated that patients with *VEGFR-1 *mRNA overexpression had shorter RFS and OS. Lymph node metastasis is generally the most important prognostic factor in breast cancer patients [12]. In this study, *VEGFR-1 *mRNA expression was shown to be associated with lymph node metastasis and to influence prognosis. In addition, multivariate analysis revealed that *VEGFR-1 *mRNA expression in peripheral blood was an independent prognostic factor, in addition to lymph node metastasis and hormone receptor status. We speculate that *VEGFR-1 *expression in the peripheral blood at the time of surgery has a higher sensitivity as a prognostic factor in breast cancer patients.

We hypothesized that the original cells expressing VEGFR-1 in breast cancer patients are monocytes, circulating endothelial cells, circulating cancer cells and hematopoietic progenitor cells (HPCs). Our previous study revealed that the original cells producing VEGFR-1 may be monocytes [10]. Jain *et al*. revealed that the frequency of peripheral blood VEGFR1^+ ^HPCs in patients with breast cancer was increased prior to relapse and could be used to predict disease progression in metastatic breast cancer patients [13]. Therefore, we have been interested in changes of VEGFR-1 mRNA in the blood of breast cancer patients before recurrence.

Recently, DNA microarray profiling has identified distinct subtypes of breast cancer. Sorlie *et al*. categorized expression profiles into five molecular subtypes: luminal A, luminal B, HER2, normal breast-like and basal-like [14]. These breast cancer subtypes are associated with different clinical outcomes, from the relatively good prognosis of patients with luminal A tumors to the worst prognosis of those with basal-like and HER2-positive tumors [15]. Ki-67 expression is chiefly important in distinguishing between 'Luminal A' and 'Luminal B (HER2 negative)' subtypes. Chemotherapy is indicated for most patients with 'Luminal B' disease [16]. Based upon the results of this study (Figure 4a), *VEGFR-1 *mRNA expression in peripheral blood may be useful for choice of adjuvant chemotherapy in Luminal type (HER2 negative) breast cancer. However, further studies are needed to analyze both Ki67 and *VEGFR-1 *expression. Basal-like breast cancer does not express estrogen receptor, progesterone receptor and HER2 (so called triple-negative breast cancer). Therefore, the only treatment available for basal-like cancer patients is systemic chemotherapy. Basal-like cancer is associated with an aggressive clinical course, distant metastasis (for example, to the brain, liver, lung and bone), and poor prognosis [17,18]. The results of the present study indicate that patients with *VEGFR-1 *-positive triple-negative cancer had a particularly poor prognosis (Figure 4b). Therefore, *VEGFR-1 *mRNA may be a useful marker for identifying poor prognosis in patients with triple-negative cancer. For such cases, an additional course of chemotherapy or close follow-up may be recommended.

*VEGFR-1 *expression was not significantly different in patients who were positive for HER2. The reason may be we had unknown HER2 data with some cases. Furthermore, the group positive for *VEGFR-1 *was small, and the chemotherapeutic regimen (including use of trastuzumab, the anti-HER2 antibody) was different from that used today.

Recently, molecularly targeted therapies (for example, the anti-HER2 antibody trastuzumab) have played an increasingly critical role in breast cancer treatment. Sunitinib and sorafenib are tyrosine kinase inhibitors (TKIs) with novel molecular targets, including VEGFR-1. Sunitinib is a small-molecule multi-TKI that targets multiple vascular endothelial growth factor receptors (VEGFRs), platelet-derived growth factor receptor (PDGFR), KIT and colony-stimulating factor 1 receptor [19]. In a breast cancer clinical trial, sunitinib demonstrated a benefit in 38.9% of 18 patients with previously treated metastatic breast cancer [20]. In a Phase II study, sunitinib treatment resulted in a partial response in 7 of 64 patients with heavily pretreated metastatic breast cancer [21]. However, a Phase III study of sunitinib combination with docetaxel improved response rate but did not prolong survival [22]. Sorafenib is a multi-kinase inhibitor that targets several receptor tyrosine kinases including, VEGFR-1, -2 and -3 and PDGFR-β, and serine threonine kinases including, Raf-1 and B-Raf, all shown to be involved in neovascularization and tumor progression [23,24]. In a Phase II randomized trial in breast cancer patients, the addition of sorafenib to capecitabine therapy significantly improved progression-free survival compared with capecitabine alone [25]. A large Phase III trial is currently evaluating this combination in a large number of breast cancer patients. We expect that *VEGFR-1 *might be useful as a biomarker, considering the efficacy of the TKIs described above.

## Conclusions

The evaluation of *VEGFR-1 *mRNA in the peripheral blood of breast cancer patients may play a very important role in the prediction of cancer metastasis and recurrence.

## Abbreviations

BMDCs: bone marrow derived cells; CI: confidence interval; CTCs: circulating tumor cells; DTCs: disseminated tumor cells; HPCs: hematopoietic progenitor cells; OS: overall survival; PDGFR: platelet-derived growth factor receptor; RFS: relapse-free survival; RPS27A: Ribosomal protein S27a; RR: relative risk; RT: reverse transcriptase; TKIs: tyrosine kinase inhibitors; VEGFR-1: vascular endothelial growth factor receptor-1

## Competing interests

The authors declare that they have no competing interests.

## Authors' contributions

YK carried out the RT-PCR analysis, statistical analysis and wrote the manuscript. AK, HY and SO contributed clinical information. HU, SA, NS and MK participated in the interpretation of data and the critical review of the manuscript. MW, KM and MM wrote parts of the article and revised it. All authors read and approved the final manuscript.
